# Mobility is Associated with Higher-risk Sexual Partnerships Among Both Men and Women in Co-resident Couples in Rural Kenya and Uganda: A Longitudinal Cohort Study

**DOI:** 10.1007/s10461-022-03878-0

**Published:** 2022-11-01

**Authors:** Sarah A. Gutin, Torsten B. Neilands, Edwin D. Charlebois, Monica Getahun, Jaffer Okiring, Adam Akullian, Irene Maeri, Patrick Eyul, Sarah Ssali, Craig R. Cohen, Moses R. Kamya, Elizabeth A. Bukusi, Carol S. Camlin

**Affiliations:** 1 Center for AIDS Prevention Studies, Division of Prevention Science, Department of Medicine, University of California, San Francisco (UCSF), 550 16th Street, 3rd Floor, 94143 San Francisco, CA, USA; 2 Department of Obstetrics, Gynecology & Reproductive Sciences, University of California, San Francisco (UCSF), Oakland, CA, USA; 3 The Infectious Diseases Research Collaboration, Kampala, Uganda; 4 Institute for Disease Modeling, Bill & Melinda Gates Foundation, Seattle, WA, USA; 5 Centre for Microbiology Research, Kenya Medical Research Institute, Nairobi, Kenya; 6 School of Women and Gender Studies, Makerere University, Kampala, Uganda; 7 Department of Medicine, School of Medicine, Makerere University College of Health Sciences, Kampala, Uganda

**Keywords:** Mobility, Higher-risk sexual partnerships, Couples, Kenya: Uganda

## Abstract

Population mobility is associated with higher-risk sexual behaviors in sub-Saharan Africa and is a key driver of the HIV epidemic. We conducted a longitudinal cohort study to estimate associations between recent mobility (overnight travel away from home in past six months) or migration (changes of residence over defined geopolitical boundaries) and higher-risk sexual behavior among co-resident couples (240 couples aged ≥ 16) from 12 rural communities in Kenya and Uganda. Data on concurrent mobility and sexual risk behaviors were collected every 6-months between 2015 and 2020. We used sex-pooled and sex-stratified multilevel models to estimate associations between couple mobility configurations (neither partner mobile, male mobile/female not mobile, female mobile/male not mobile, both mobile) and the odds of higher-risk (casual, commercial sex worker/client, one night stand, inherited partner, stranger) and concurrent sexual partnerships based on who was mobile. On average across all time points and subjects, mobile women were more likely than non-mobile women to have a higher-risk partner; similarly, mobile men were more likely than non-mobile men to report a higher-risk partnership. Men with work-related mobility versus not had higher odds of higher-risk partnerships. Women with work-related mobility versus not had higher odds of higher-risk partnerships. Couples where both members were mobile versus neither had greater odds of higher-risk partnerships. In analyses using 6-month lagged versions of key predictors, migration events of men, but not women, preceded higher-risk partnerships. Findings demonstrate HIV risks for men and women associated with mobility and the need for prevention approaches attentive to the risk-enhancing contexts of mobility.

## Introduction

Population mobility, which includes both international and internal migration, and complex, localized, and shorter-term forms of mobility, is a major driver of the HIV epidemic in sub-Saharan Africa (SSA) [1–6]. Mobile individuals tend to engage in higher-risk sexual behaviors [2, 3, 7, 8], and are at greater risk of HIV acquisition and transmission [4–6].

Though much attention has focused on the HIV risk among male migrants, women tend to migrate at rates exceeding those of men in the region [3]. While men are more mobile than women overall [8], when women are the members within couples who migrate, they have been found to do a higher number of trips and to spend more nights away from home compared to men, heightening their HIV acquisition risks [2, 8, 9]. While associations between mobility and higher-risk sexual partnerships that differ by sex have been noted cross-sectionally at the individual-level [8], for individuals and couples, it is unclear whether different forms of mobility (work or non-work related mobility, migration) affect the probability of higher-risk partnerships over time.

Previous studies of mobility and sexual risk behavior have been subject to some methodological shortcomings and measurement challenges. The effects of mobility are usually examined at the individual level without considering the dependent nature of couple-level data [10]. Mobility in one partner can influence the sexual behavior of the other partner, whether they are mobile or not [11, 12]. In some cases being separated from one’s spouse is associated with reduced sexual risk behavior [13], while in other situations the risk for the female partners of male migrants is either increased [12] or unchanged [2, 6, 14]. In addition, the temporal dimensions of population mobility are important, yet few studies have accounted for characteristics of the dyad within a longitudinal design to establish associations or examined higher-risk behaviors by the type of mobility [15, 16]. Also, little is known about how gender and couple characteristics influence mobility and behavioral risks for HIV over time since longitudinal studies among couples are rare.

To overcome previous research gaps, we leveraged data from a community randomized controlled test-and-treat study in eastern Africa, and utilized couple mobility configuration effects (neither partner mobile, male mobile/female not mobile, female mobile/male not mobile, both mobile) and sex-stratified analyses to investigate the relationship between mobility and sexual behaviors. In these analyses, we used prospective, longitudinal dyadic data as well as high-resolution measures of mobility and sexual behavior to: (1) examine the individual’s influence of mobility on engaging in higher-risk sexual partnerships and evaluate whether this differs by sex, as well as (2) test for associations between couple mobility configurations and higher-risk and concurrent sexual partnerships. Our results have implications for designing interventions specific to mobile men, women, and couples.

## Methods

### Study Design and Participants

Data are from a prospective cohort study of mobility (R01MH104132) in communities [8] participating in the Sustainable East Africa Research in Community Health (SEARCH) trial (NCT01864603) [17]. SEARCH examined the impact of treatment-as-prevention (TasP) in 32 communities in western Kenya and eastern and southwestern Uganda (HIV prevalence of 10% overall) [17]. The mobility study was conducted in 12 SEARCH communities (HIV prevalence of 13.7% overall) [8].

We enrolled 480 HIV-negative adults at baseline, aged ≥ 16 (i.e. a couples-based random sample of 240 pairs of male and female participants) with a consent rate of 98.3% to derive the cohort. A multi-level stratified random sampling design (region, sex, and mobility status at baseline [i.e. both members of couple are mobile, male mobile and female not, female mobile and male not, both not mobile]) was used to select the sample of couples from the census-enumerated adult population of each of 12 SEARCH communities, purposively selected to reflect underlying heterogeneity in forms of mobility. Baseline mobility status was defined as being away from the household for 6 months or more in past year and/or fewer than half of nights spent in the household in past 4 months. Couples from each community were selected by mobility status to include 5 couples where both male and female were mobile, 5 couples where both male and female were non-mobile, 5 couples where the male was mobile and the female non-mobile, and 5 couples where the male was non-mobile and the female was mobile. There was some loss to follow-up over time but at the final round of data collection, 94% of the sample was retained (round 1 n = 480, round 2 n = 467, round 3 n = 464, round 4 n = 465, round 5 n = 461, round 6 n = 462, round 7 n = 453, round 8 n = 451).

### Procedures

Mobility and sexual risk behaviors survey data were collected during a baseline visit between February-November 2016 and followed by one study visit every 6 months thereafter until February 2020. Therefore, participants were surveyed at up to eight time points (eight rounds of data collection conducted every 6 months). Survey data were collected using programmed tablets and took about 90 min to complete; topics included demographics, migration histories, work and non-work related mobility in past six months, and sexual risk behaviors.

Previously collected SEARCH data (the dataset on which the sample was constructed) were used to populate socio-demographic factors including age, education, household wealth, and occupational risk. Some data such as age and marital status were reconfirmed during data collection. Household wealth was divided into the lowest wealth quintile versus all other wealth quintiles. Occupation types were originally grouped into the categories informal sector low-risk (e.g., farming/livestock, student, construction/artisanal labor, shopkeeper/market vendor, household worker/housewife), formal sector low-risk (e.g., government/military, teacher, healthcare, factory worker/mining) and informal sector high-risk (e.g., fishing/fish trade, hotel/restaurant/bar worker, transport/tourism) but were collapsed in models into “informal/formal low-risk” and “informal high-risk” categories on the basis of underlying HIV prevalence levels within those livelihood categories.

The baseline mobility survey captured participants’ histories of migrations over their lifetime by asking participants to tell us their birthplace and the names of places they lived (with county/district/nation recorded by the interviewer) along with their age at change of residence in childhood to the present. Migration was defined as movement of people across a specified geopolitical boundary (nation, district and sub-county) for the purpose of establishing a new permanent residence. Migration between countries was classified as international; migration within countries as internal migration.

Interviewers also asked about mobility in the past six months before the study visit (recorded in six month intervals), including labor-related and non-labor-related mobility that required sleeping away from the main residence. As described elsewhere [1], interviewers collected detailed data on the names of all locations where participants travelled, including county/district, number of trips, and number of nights per trip. Mobility was defined as travel involving time spent away from primary places of residence, without any intention to change residence (locations and movements between multiple homes that are considered to be main residences were also recorded). This excluded commuting, as mobility is recorded only if the travel involved sleeping one or more nights away from primary residence(s). Labor-related mobility was defined as travel “for business/to earn money”, including travel to look for a job and for farming/food production. Non-labor-related mobility was defined as travel for all other purposes. Mobility was measured in each six-month interval.

We adapted a detailed calendar-based data collection tool [8] to collect sexual and behavioral histories for sexual partnerships since January 2011. This tool was informed by a relationship history calendar [18] previously used in the region [8] and shown to reduce social desirability bias to improve the reporting of sexual relationships and behavior [18]. The survey records information in monthly intervals rather than years because many relationships last less than a year; we measured changes in relationship dimensions and behaviors over the course of each sexual relationship in the preceding five years from the time of the baseline survey (i.e., since January 2011). The calendar was used to collect monthly data on partnerships, including relationship type, mobility of partners, and partner concurrency. For the partner they were living with and for all additional partners, we asked about months when there was sexual activity with each partner. So, it is possible that a member of the index partnership said that there was no sex in the index relationship in a given month, but they might have reported sex with a different partner in that month. If the sex in these two relationships overlapped, then there was concurrency in the time period. Our measures allowed us to accurately measure concurrency without asking directly about the total number of partners in a given month. If the sex did not overlap in the same month, then it was not considered to be concurrent. Aggregate measures of higher-risk sexual partnerships and relationship concurrency were developed for each six-month interval.

We created two designations for higher-risk sexual partnerships: (1) relationships that were casual, commercial sex worker/client, “one night stand”, stranger, or inheritor/inherited partner, and (2) higher-risk including concurrent sexual partnerships (where reports of concurrency were in addition the higher-risk relationships as defined above). We created this second designation including concurrent sexual partnerships because there were few women who fell into the higher-risk category in each round. Including concurrent partnerships in this designation offered the ability to run models for women with higher outcome numbers while still preserving the intent of the higher-risk designation as it pertains to women, for whom concurrent partnerships are more rare and indicative of sexual risk, compared to men, for whom concurrent partnerships are more common and carry less stigma. Inherited partner refers to the traditional practice of *widow inheritance* in which a woman is expected to engage in sex with another man following the death of her husband to fulfill certain spiritual requirements [3]. Traditionally, the widow is “inherited” by another man in the husband’s family so that children are retained within the patrilineage and the widow and her children are provided for. However, the practice has influenced the spread of HIV in the region [3].

### Ethical Approval

Ethical approvals were received from the University of California San Francisco Committee on Human Research (14–15058), Ethical Review Committee of the Kenya Medical Research Institute (KEMRI/SERU/CMR/3052), Makerere University School of Medicine Research and Ethics Committee (2015–040), and Uganda National Council for Science and Technology (HS 1834).

### Statistical Analysis

Statistical analyses were used to describe population characteristics. Bivariate comparisons that accounted for clustering of individuals within dyads (Rao-Scott F-tests) were used to characterize the relationship between higher-risk sexual partnerships and mobility. Longitudinal multilevel logistic models that estimated random intercepts and slopes and their covariance for person ID with cluster adjustment for dyad ID were fitted using Stata statistical software (version 16.1) to examine contemporaneous associations between recent mobility or migration with higher-risk sexual partnerships or higher-risk and concurrent sexual partnerships. Models were adjusted for time, age, education, occupational risk status, and household wealth. We opted for a random-effects model because several of the covariates were time constant (and fixed-effects models cannot incorporate time-invariant variables) and because theoretically, and based on the literature, those variables were important to include as confounders in the models. In addition, as a sensitivity analysis, due to small numbers of positive responses at some time points, we refitted models using penalized maximum likelihood estimation methods [19]. Confidence intervals for penalized models were generated via cluster bootstrapping based on 5,000 bootstrap samples. We report bias-corrected (BC) bootstrap confidence intervals. We explored a time-by-exposure interaction, to test whether mobility effects on the outcomes varied across time. We compared models with and without interaction using the BIC statistic and for all models, the BIC favored the models without interaction. We therefore report the results from the models without interaction. Finally, as an additional sensitivity analysis, we reran each model substituting 6-month lagged versions of the predictors for the contemporaneous predictors used in the main models. Models which failed to converge with random slopes and intercepts were refitted with random intercepts only to attain convergence.

## Results

Table I describes the baseline characteristics of the study population and their mobility and sexual behaviors by sex and adjusted for clustering. Male participants were older than female participants on average (45 vs. 37 years old, F(1, 239) = 331.16, p < 0.001). All couples were married and had been in relationships for about 19.5 years. The majority of participants had either no schooling or some primary-level education (78.6%). More men than women had completed primary education or received some higher-level education (28.5% vs. 14.3%, F(1, 239) = 24.95, p < 0.001). Overall, more participants were involved in informal sector or formal sector low-risk work (87.2%) compared to informal sector high-risk work (12.9%) with more men than women involved in informal sector high-risk work (17.0% vs. 8.6%, F(1.97, 467.47) = 6.19, p = 0.002).

At baseline, men were more likely than women to engage in labor-related mobility in the past 6 months (23.8% vs. 1.3%, F(1, 239) = 56.01, p < 0.001) while women were more likely than men to engage in non-labor-related mobility in the past 6 months (52.1% vs. 35.0%, F(1, 239) = 19.34, p < 0.001). These relationships were similar within the past 1-month as well. Overall, men and women reported equal numbers of migrations within the past 6 months (2.5% each). In the past 6 months, 52.1% of participants reported some type of mobility with men and women reporting almost equal frequencies of mobility (51.7% vs. 52.5%, respectively). Across all eight rounds of data collection, there were 358 reports of work related mobility by men and 40 reports of work-related mobility among women (Round 1 = 3 women reporting work-related mobility in the past 6 months, Round 2 = 5 women, Round 3 = 3 women, Round 4 = 5 women, Round 5 = 4 women, Round 6 = 4 women, Round 7 = 9 women, and Round 8 = 7 women) from 21 unique women (data not shown).

A higher proportion of men (22.5%) than women (4.6%) reported sexual partnership concurrency in the past 6 months (F(1, 239) = 34.79, p < 0.001). In addition, 4% of individuals in the study reported partnerships classified as higher-risk, with men (5.8%) reporting higher-risk partnerships more than women (2.1%) in the past 6 months (F(1, 239) = 4.80, p = 0.030). Across all eight rounds of data collection, there 77 reports of higher-risk sexual partners by men and 85 reports of higher risk sexual partners among women (Round 1 = 5 higher-risk partnerships reported by women, Round 2 = 10 women, Round 3 = 12 women, Round 4 = 12 women, Round 5 = 13 women, Round 6 = 11 women, Round 7 = 9 women, and Round 8 = 13 women) from 21 unique women (data not shown).

**Fig. 1:**
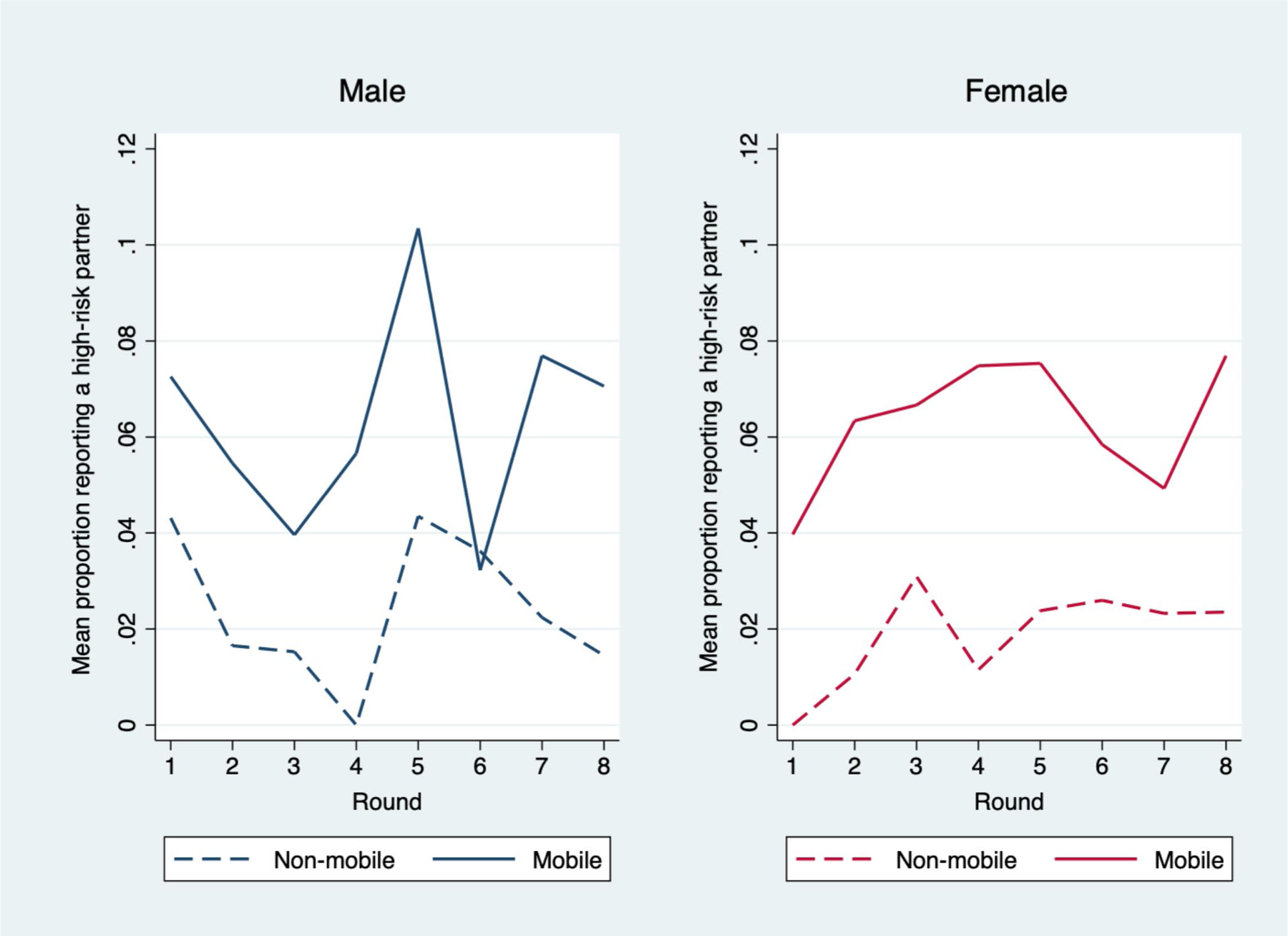
Mean proportion of mobile and non-mobile men and women reporting a higher-risk partner by data collection round (2015–2020)

Table II describes higher-risk partnerships of men and women by mobility status. On average across all time points and all subjects, mobile women (6.3%) were more likely than non-mobile women (1.8%) to have a higher-risk partner (F(1, 239) = 15.11, p < 0.001); similarly, on average across all time points, mobile men (6.4%) were more likely than non-mobile men (2.4%) to report a higher-risk partnership (F(1, 239) = 15.99, p < 0.001). Table II also describes higher-risk/concurrent partnerships by mobility status and sex. On average across all time points and all subjects, mobile women (9.9%) were more likely than non-mobile women (2.8%) to have a higher-risk/concurrent partnership (F(1, 239) = 23.99, p < 0.001); similarly, on average across all time points and all subjects mobile men (34.5%) were more likely than non-mobile men (26.4%) to report a higher-risk/concurrent partnership (F(1, 239) = 4.58, p = 0.033). Age adjusted results are presented in Table II. Over time, mobile men had a higher mean proportion of higher-risk partners than non-mobile men and mobile women had a higher mean proportion of higher-risk partners than non-mobile women (Fig. 1).

Table III-VII show adjusted odds ratios from longitudinal multilevel models fitted to examine associations between types of mobility on higher-risk sexual partnerships. In sex-stratified models, relative to non-mobile men, men who reported any mobility had higher odds of higher-risk partnerships (aOR = 2.14, 95%CI: 1.10–4.19, p = 0.014) (Table III). Additionally, relative to men who did not engage in work-related mobility, men who were mobile for work had higher odds of higher-risk partnership (aOR = 2.15, 95%CI: 1.03–4.48, p = 0.043) (Table III). Male models examining the effects of non-work related mobility and migration were non-significant. Further, relative to women who did not engage in work-related mobility, women who were mobile for work had higher odds of higher-risk partnerships (aOR = 23.65, 95%CI: 2.11–265.41, p = 0.011) and sensitivity analyses performed with penalized logistic regression found similar results to the main model (aOR = 5.10, 95%CI: 1.14–15.77, p = 0.014) (Table IV). Female models examining the effects of non-work related mobility, migration, and overall mobility were non-significant.

In couple-level models, when both the male and female partners were mobile at the same time, there were higher odds of higher-risk partnerships and higher odds of higher-risk/concurrent partnerships (Supplemental Table I). Similarly, in sex stratified models, relative to couples where neither member was mobile, men had higher odds of higher-risk partnerships if both they and their partner were mobile and men had higher odds of higher-risk partnerships if they were mobile for work-related reasons without their female partner (Supplemental Table II). Couple models examining the effects of non-work related mobility and migration on men were non-significant and couple models examining the effects of overall mobility, work and non-work related mobility, and migration on women were all non-significant.

In sensitivity analyses using 6-month lagged versions of the key predictors, we found differences by sex in the types of mobility associated with higher-risk partnerships. In lagged sex-stratified models, while female models examining the effects of work-related mobility, non-work related mobility, migration, and any mobility overall were all non-significant, the male model for migration was significant (aOR = 4.59, 95%CI: 1.002–21.03, p = 0.050), showing that migration events preceded higher-risk partnerships in men (Table V). Lagged male models examining the effects of work-related mobility, non-work related mobility, and any mobility overall were non-significant.

In lagged couple-level models, if there was any mobility, and both members of the couple were mobile, there were increased odds of higher-risk partnership (aOR = 1.95, 95%CI: 1.01–3.79) compared to relationships in which neither partner was mobile (Table VI). This shows that when both couple members are mobile, that mobility preceded higher-risk partnerships. Lagged couple-level models examining the effects of work and non-work-related mobility, and migration were non-significant.

In lagged sex-stratified couple models, if women were mobile for work but men were not, men had higher odds of higher-risk partnerships compared to relationships where neither couple member was mobile (aOR = 7.02, 95%CI = 1.46–33.74, p = 0.015) (Table VI). In addition, if men had migrated but women had not, then the men had higher odds of higher-risk partnerships compared to relationships where neither couple member had migrated (aOR = 9.20, 95%CI = 1.85–45.81, p = 0.007) (Table VII). This again showed that male migration preceded higher-risk partnerships. Sex-stratified couple models for any mobility and non-work related mobility in men were not significant. Additionally, in lagged sex-stratified couple models, if men had migrated but women had not migrated, women had lower odds of a higher-risk or concurrent partnerships (aOR = 0.05, 95%CI = 0.004–0.65, p = 0.022) compared to relationships where neither member of the couple had migrated (Table VII). Sex-stratified couple models for any mobility, work-related mobility, and non-work related mobility in women were not significant.

## Discussion

The findings of this longitudinal couples cohort study in Kenya and Uganda show that while some forms of mobility seemed to coincide in the same time period with higher-risk partnerships (such as work-related mobility in men and women), migration in particular was different, and the migration events of men, but not women, clearly preceded higher-risk partnerships. In addition, while recent work-related mobility was significantly associated with contemporaneous higher-risk partnerships among both men and women, the risks appear more pronounced for women. These findings highlight the gendered impact of mobility on sexual risk behaviors that are revealed through examining the mobility and sexual behaviors of couples.

Studies in sub-Saharan Africa (SSA) have noted that mobile men are at an increased risk for HIV and engage in higher-risk sexual behaviors [10, 20, 21]. In addition, work-related mobility in men has been associated with higher-risk partnerships such as concurrent relationships [3, 8, 20]. The finding in this study that men in co-habiting couples who experienced recent mobility or engaged in work-related mobility had a higher probability of higher-risk partnerships supports these previous findings. In addition, a time by mobility interaction was non-significant, indicating that this relationship was consistent over time. While most services are currently offered at fixed location clinics that mobile men find hard to access [22, 23], interventions for mobile men that facilitate HIV care engagement and flexible access to services are needed. Community-based models that meet mobile men where they are (e.g. transit hubs, popular migration destinations) may aid in accommodating the needs of mobile men [15].

Work-related mobility was also associated with higher-risk partnerships in couples, but the effect appears more pronounced among women than men. This finding among women should be considered exploratory, in light of low variability among women who reported work-related mobility. However, previous research has similarly found that the effect of mobility on HIV risk behaviors is more pronounced among women and that work-related mobility is associated with higher-risk partnerships in women [2, 3, 8]. Overall, female migrants exhibit higher risk behavior and HIV prevalence compared to non-migrant women [3]. Prior qualitative research on HIV risks among migrant and mobile women in the setting [3, 7] highlighted how women’s work-related mobility may permit them to seek opportunities to engage in transactional sex in order to supplement income while away from home communities, where their behavior is more socially monitored and subject to gender norms that restrict engagement in higher risk sexual behaviors. Our findings suggest that mobile women are in need of interventions to help them access HIV preventive care. Early identification and linkage to services for newly arrived female migrants at popular destinations may reduce HIV incidence and transmission since they are particularly vulnerable to HIV infection [21] and may engage in higher-risk sexual behaviors such as transactional sex or commercial sex work to subsist [3].

A contribution of this research is the ability to look at different couple mobility configurations (both mobile, male mobile/female not mobile, male not mobile/female mobile, neither partner mobile) and examine the odds of having a higher-risk partnership over time. While questions remain about how women’s mobility affects their non-mobile male partners, we see that when both partners are mobile, men have over two times the odds of reporting higher-risk behaviors. Data from various SSA contexts have found that the mobility of one partner can impact the HIV risk behaviors of the other [2, 11, 12]. For example, among Kenyan fishermen and their spouses, mobile women who had non-mobile spouses had 2.1 times the likelihood of HIV infection compared to individuals in couples where both partners were non-mobile, showing the mobility of spouses was associated with HIV infection that was not evident among the fishermen [2]. Couples-based approaches that address relationship dynamics (e.g., trust, couple communication) have shown promise in this context and with other mobile populations [24, 25] and have the potential to reduce higher-risk sexual behaviors and increase engagement with HIV preventive services. Future analyses utilizing a specialized dyadic analytic approach such actor-partner analysis will be needed to further examine the separate question of whether and how partners specifically affect each other’s behavior.

The longitudinal nature of this dataset also permits us to examine whether exposures precede outcomes. In lagged analyses, it was possible to see that higher-risk partnerships were more likely to follow after migration events in men, but not women, when both members of the couple were mobile, and that men tended to have higher-risk partnerships when women in a couple but not their male partners were mobile for work. Therefore, some types of mobility events, in particular migration, preceded higher-risk partnerships. It is not clear why migration events for men but not women predict future risk-events. In this context, men tend to migrate for work, while women tend to migrate for non-work related reasons. These different types of migration may open them up to different levels of risk. A certain predisposition to risk-taking could also predict higher-risk sexual behaviors and migration events. HIV status itself may also precipitate migration. While it is not clear why migration would predict future risk events for men and not women, the stronger signal in men may reflect gendered social norms, where men’s engagement in higher-risk sexual behaviors is more normative than women’s. Generally speaking, when we compare men and women, men are more likely to engage in higher-risk sexual behaviors than women and that is especially the case when they are migrating. Therefore, we would expect that male migrants engage in more higher-risk sexual behaviors compared to female migrants.

Other types of mobility, such as work-related mobility in men, tended to coincide more closely in time with higher-risk partnerships. These findings provide evidence for a causal pathway linking mobility to HIV acquisition and transmission, via higher-risk sexual behavior both subsequent to and contemporaneous with mobility events [9]. While we cannot rule out an unmeasured “predisposition to risk taking” that may precede both mobility and sexual risk behaviour, the findings underscore the importance of focusing on the mobility of populations as a crucial means to ending the HIV epidemic.

Mobility poses a significant and continued threat to HIV prevention efforts. Targeted and creative interventions, new policies, and health systems improvements are needed to fully engage mobile individuals in HIV care and prevention. At the policy level, a focus on hotspots based on high HIV prevalence has predominated, but this approach does not account for data that suggests that migrants with HIV may selectively move into low prevalence areas, and not only ‘hotspots’ [26, 27]. The movement of newly infected individuals in and out of communities can slow efforts to reduce HIV incidence [21], and to increase ART coverage and population-level viral suppression [28]. Pre-exposure prophylaxis (PrEP) roll-out for HIV-uninfected persons is also challenged by mobility, with suboptimal PrEP engagement found among mobile populations [9]. Therefore, studies that engage mobile populations and consider their needs in the design and implementation phases are urgently needed.

The design of this study confers methodological strengths compared to prior studies, which have predominantly been cross-sectional [10]. The study design allowed for the examination of different couple mobility configurations over time. This is one of the few if not only studies that has used a longitudinal design and captured behavior change along with mobility within couples over a 5-year period. Retention was very high (overall loss-to-follow-up (LTFU) across 8 rounds of data collection was 3.33% overall, 4.17% in Uganda and 2.5% in Kenya) and LTFU between rounds was no greater than 2.5% and at some rounds there was no LTFU. The study is further strengthened by the use of high-resolution measurements of mobility, partnerships, and sexual risk behaviors. For example, our relationship history calendar instrument was carefully designed to accurately measure concurrency and is structured in such a way that we do not have to directly ask about the total number of partners in a given month. Instead, we ask about all relationships and then can accurately see when partnerships overlapped. This approach is less subject to social desirability bias. This is a rigorous measure and the estimates of concurrency from our data are higher than in other datasets/studies. Overall, in our sample, 22.5% of men and about 5% of women at baseline reported sexual concurrency while in the most recent Kenya Demographic and Health Survey, less than 1% of women, and 5% of men reported sexual partner concurrency six months before the survey [29]. We believe our innovative data collection methods produce a more accurate measure. However, in future research, aspects of mobility such as number of trips, days spent away, and locations travelled may be an important line of investigation.

This study was subject to limitations. Despite comprehensive measures being used repeatedly every six months, demographic and sexual history data were self-reported and it is possible that social desirability or recall bias occurred. However, social desirability bias was minimized in several ways (stressing confidentiality, interviewing men and women with sex-matched interviewers, follow-up interactions conducted by the same research assistants to establish rapport/comfort, prompts that normalized behaviors such as multiple partnerships, and asking about each partner separately so that questions do not directly ask about relationship concurrency but can be derived from the dataset). Since measures only asked about behaviors within the past 6 months, recall bias should also be minimized. Some of our analyses involving women have small numbers and so the results should be considered more exploratory. However, the low variability among women does not negate the reporting of these results and still provides fine grained, rich, and novel findings that are worthy of reporting. We recommend that future studies collect more data from women specifically to delve into their specific concerns more fully. This was a balanced sample and so the results likely do not represent risk levels in the general population and the results may not be generalizable to other settings. The SEARCH sample was believed to be composed of stable residents (defined as those who spent at least 6 months of the previous year in the trial community) [17], and yet we saw that population mobility was pervasive, similar to other rural African contexts [8, 30, 31]. While the HIV incidence in SEARCH was low (10% overall) and declined further during the trial period due to universal testing and linkage to care, within this sample of 12 communities, the number of sero-conversions was too small to statistically test. Although the outcome of HIV incidence is of key clinical importance, this study was not powered to detect differences in HIV incidence.

In conclusion, work-related mobility is associated with higher-risk partnerships among both men and women in Kenya and Uganda but the effect for women is more pronounced. In addition, if both partners are mobile, the odds of higher-risk sexual partnerships among men are further exacerbated. In lagged analyses that establish temporally-ordered pathways, higher-risk partnerships were more likely to follow after migration events in men, but not women. These findings highlight the HIV risks for men and women associated with mobility and the added risk of female partner mobility for men. Traditional HIV prevention approaches do not adequately engage mobile populations, a key target if we hope to end the HIV epidemic. Interventions that prioritize the needs of mobile populations and address the particular challenges they face are urgently needed as these populations face barriers to treatment and care. A combination of sex-specific and couple-based HIV prevention programs that are mobility and migration-aware are warranted.

## Supplementary Material

Suppl Table 1

Suppl Table 2

## Figures and Tables

**Table I:** Socio-demographic, mobility and sexual risk behavior characteristics by sex at baseline, adjusted for clustering

Characteristic	Category	Overall		Sex		

		n = 480	Men	Women	F-test (df)	p-value*
**Age**	Mean Age (SE)	41.26 (0.71)	45.16 (1.02)	37.37 (0.91)	F (1, 239) = 331.16	**< 0.001**
**Region**	Kenya - Western	240 (50.0)	120 (50.00)	120 (50.00)	F (1, 239) = < 0.001	1.000
	Uganda - Eastern	120 (25.0)	60 (25.00)	60 (25.00)		
	Uganda - South Western	120 (25.0)	60 (25.00)	60 (25.00)		
**Marital Status**	Currently married	480 (100.0)	240 (100.0)	240 (100.00)	-	-
**Relationship Length**	Mean relationship length in years (SE)	19.55 (0.67)	19.51 (0.91)	19.60 (0.95)	F (1, 232) = 0.02	0.892
	Median relationship length in years (IQR)	16 (9–26)	16 (9–25)	16 (9–27)	-	-
**Education level**	No Schooling + some primary	374 (78.57)	170 (71.43)	204 (85.71)	F (1, 239) = 24.95	**< 0.001**
	Completed primary and higher	102 (21.43)	68 (28.57)	34 (14.29)		
**Household wealth**	Poorest wealth quintile	62 (12.92)	31 (12.92)	31 (12.92	-	-
	All other wealth quintiles	418 (87.08)	209 (87.08)	209 (87.08)		
**Occupation**	Informal sector *(low risk, e.g.: farming/livestock, student, construction/artisanal labor, market vendor/shopkeeper, household worker/housewife)*	383 (82.01)	181 (77.02)	202 (87.07)	F (1.97, 467.47) = 6.19	**0.002**
	Formal sector *(low risk, e.g.: Government/military/teacher/healthcare, factory worker/mining)*	24 (5.14)	14 (5.96)	10 (4.13)		
	Informal sector *(high risk, e.g.: fishing/fish trade, hotel/restaurant/bar worker, transport/tourism)*	60 (12.85)	40 (17.02)	20 (8.62)		
**Short-term Mobility**	*Past 6 months mobility ( > = 1 nights away)*					
	Any labor-related mobility past 6 mo.	60 (12.50)	57 (23.75)	3 (1.25)	F (1, 239) = 56.01	**< 0.001**
	Any non-labor-related mobility, past 6 mo.	209 (43.54)	84 (35.00)	125 (52.08)	F (1, 239) = 19.34	**< 0.001**
	*Past 1 month mobility ( > = 1 nights away)*					
	Any labor-related mobility, past 1 mo.	33 (6.88)	31 (12.92)	2 (0.83)	F (1, 239) = 28.72	**< 0.001**
	Any non-labor-related mobility, past 1 mo.	103 (21.46)	38 (15.83)	65 (27.08)	F (1, 239) = 9.90	**0.002**
**Any migration**	Any inter/intra-district migrations in past 6 mo.					
	No	468 (97.50)	234 (97.50)	234 (97.50)	F (1, 239) = 1.09	0.299
	Yes	12 (2.50)	6 (2.50)	6 (2.50)		
**Any mobility**	Any mobility (work/non-work mobility or migrations) in past 6 mo.					
	No	230 (47.92)	116 (48.33)	114 (47.50)	F (1, 239) = 1.09	0.832
	Yes	250 (52.08)	124 (51.67)	126 (52.50)		
**Sexual concurrency**	Any concurrent sex partnerships, past 6 months	65 (13.54)	54 (22.50)	11 (4.58)	F (1, 239) = 34.79	**< 0.001**
**Higher-risk sex partners**	Any higher-risk sex partners, past 6 mo.	19 (3.96)	14 (5.83)	5 (2.08)	F (1, 239) = 4.80	**0.030**

* Cluster-adjusted design-based Rao-Scott F test

**Table II:** Higher-risk partnerships and higher-risk and concurrent partnerships by mobility status averaged across all time points and all subjects, stratified by sex and adjusted for couple-level clustering

Characteristic	Men (n = 1844)					Women (n = 1859)				
	Not mobile	Mobile	F-test (df)	*F-test p-value*	*Age adjusted p-value*	Not mobile	Mobile	F-test (df)	*F-test p-value*	*Age adjusted p-value*
Any higher risk sex partners during follow-up										
No	994 (97.64)	773 (93.58)	F (1, 239)	***< 0.001***	***0.017***	711 (98.20)	1063 (93.66)	F (1, 239)	***< 0.001***	***0.012***
Yes	24 (2.36)	53 (6.42)	= 15.99			13 (1.80)	72 (6.34)	= 15.11		
Any higher risk /concurrent sex partners during follow-up										
No	749 (73.58)	541 (65.50)	F (1, 239)	***0.033***	*0.078*	704 (97.24)	1023 (90.13)	F (1, 239)	***< 0.001***	*0.089*
Yes	269 (26.42)	285 (34.50)	= 4.58			20 (2.76)	112 (9.87)	= 23.99		

**Table III:** Association of metrics of mobility (for any purpose, and for work) with higher-risk sexual partnerships in men, 2016–2020 (n = 1793)

Variable	Category	Men: ANY mobility			Men: Work-related mobility		

		aOR	95% CI	*p-value*	aOR	95% CI	*p-value*
Time	Round	0.83	0.60–1.14	0.263	0.83	0.60–1.16	0.270
Age	Mean age	0.99	0.96–1.02	0.475	0.99	0.96–1.02	0.597
Education	Ref: No education or some primary	-	-	-	-	-	-
	Completed primary and higher	0.89	0.35–2.31	0.821	0.92	0.33–2.59	0.873
Occupation*	Ref: Formal and informal sector low-risk	-	-	-	-	-	-
	Informal sector high-risk	2.00	0.71–5.66	0.177	2.26	0.82–6.29	0.116
Household wealth	Ref: All other quartiles	-	-	-	-	-	-
	Poorest quartile	2.66	0.87–8.10	0.087	2.52	0.79–8.05	0.118
Mobility	Ref: No mobility in past 6 mo.	-	-	-	-	-	-
	Any mobility in past 6 mo.	2.14	1.10–4.19	**0.014**	2.15	1.03–4.48	**0.043**

In these models, mobility measures any mobility (left) and work-related mobility (right).

* Occupational risk categories were collapsed into two categories (informal/formal low-risk and informal high-risk)

**Table IV:** Association of work-related mobility with higher-risk and concurrent sexual partnerships in women, 2016–2020 (n = 1780)

Variable	Category	Work-related mobility					

		Multi-level model			Penalized maximum likelihood estimation with cluster bootstrapping		

		aOR	95% CI	*p-value*	aOR	95% CI	*p-value*
Time	Round	1.37	0.85–2.22	0.195	1.06	0.98–1.15	0.147
Age	Mean age	0.96	0.92–1.00	0.080	0.97	0.93–1.00	0.091
Education	Ref: No education or some primary	-	-	-	-	-	-
	Completed primary and higher	1.47	0.25–8.74	0.668	0.98	0.14–3.55	0.980
Occupation*	Ref: Formal and informal sector low-risk	-	-	-	-	-	-
	Informal sector high-risk	6.15	1.30–28.98	0.022	2.15	0.36–6.71	0.306
Household wealth	Ref: All other quartiles	-	-	-	-	-	-
	Poorest quartile	1.09	0.21–5.57	0.917	1.27	0.12–3.99	0.781
Work-related mobility	Ref: No work-related mobility, past 6 mo.	-	-	-	-	-	-
	Work-related mobility in past 6 mo.	23.65	2.11–265.41	**0.011**	5.10	1.14–15.77	**0.014**

In these models, mobility measures work-related mobility.

* Occupational risk categories were collapsed into two categories (informal/formal low-risk and informal high-risk)

**Table V:** Lagged association of migration with higher-risk sexual partnerships in men, 2016–2020 (n = 1793

Variable	Category	Men: ANY mobility		

		aOR	95% CI	*p-value*
Time	Round	0.65	0.44–0.94	0.023
Age	Mean age	0.99	0.97–1.01	0.447
Education	Ref: No education or some primary	-	-	-
	Completed primary and higher	0.99	0.43–2.27	0.975
Occupation*	Ref: Formal and informal sector low-risk	-	-	-
	Informal sector high-risk	2.33	1.05–5.17	**0.038**
Household wealth	Ref: All other quartiles	-	-	-
	Poorest quartile	2.10	0.89–4.95	0.089
Migration	Ref: No migration, past 6 mo.	-	-	-
	Migration in past 6 mo.	4.59	1.002–21.03	**0.050**

* Occupational risk categories were collapsed into two categories (informal/formal low-risk and informal high-risk)

**Table VI:** Lagged association of couple-level mobility and of couple-level work-related mobility in men on higher-risk sexual partnerships, 2016–2020

Variable	Category	Sex pooled ANY mobility (n = 3500)			Men: work-related mobility (n = 1765)		

		aOR	95% CI	*p-value*	aOR	95% CI	*p-value*
Time	Round	1.02	0.92–1.14	0.665	0.66	0.46–0.95	**0.025**
Age	Mean age	0.97	0.95–0.999	**0.038**	0.99	0.97–1.01	0.426
Education	Ref: No education or some primary	-	-	-	-	-	-
	Completed primary and higher	0.74	0.32–1.71	0.473	0.94	0.41–2.18	0.891
Occupation*	Ref: Formal and informal sector low-risk	-	-	-	-	-	-
	Informal sector high-risk	3.42	1.53–7.64	**0.003**	2.06	0.87–4.85	0.099
Household wealth	Ref: All other quartiles	-	-	-	-	-	-
	Poorest quartile	2.50	0.91–6.87	0.075	1.74	0.73–4.13	0.209
Mobility	Ref: No mobility in couple, past 6 mo.	-	-	-	-	-	-
	Male mobile, female not	1.93	0.89–4.19	0.096	1.95	0.99–3.86	0.054
	Female mobile, male not	1.11	0.57–2.17	0.758	7.02	1.46–33.74	**0.015**
	Both male and female mobile	1.95	1.005–3.79	**0.048**	1.37	0.07–26.53	0.835

In these models, mobility measures any mobility (left) and work-related mobility (right).

* Occupational risk categories were collapsed into two categories (informal/formal low-risk and informal high-risk)

**Table VII:** Lagged association of couple-level migration on higher-risk sexual partnerships among men and couple-level migration on higher-risk and concurrent sexual partnerships in women, 2016–2020

Variable	Category	Men: migration (n = 1756)			Women: migration (n = 1725)		

		aOR	95% CI	*p-value*	aOR	95% CI	*p-value*
Time	Round	0.66	0.46–0.96	**0.028**	0.76	0.65–0.88	**< 0.001**
Age	Mean age	0.99	0.97–1.01	0.414	0.97	0.95–0.996	**0.023**
Education	Ref: No education or some primary	-	-	-	-	-	-
	Completed primary and higher	1.00	0.43–2.30	0.996	0.96	0.30–3.07	0.950
Occupation*	Ref: Formal and informal sector low-risk	-	-	-	-	-	-
	Informal sector high-risk	2.43	1.09–5.39	**0.029**	2.41	0.72–8.10	0.153
Household wealth	Ref: All other quartiles	-	-	-		-	**-**
	Poorest quartile	2.26	0.97–5.27	0.059	1.15	0.39–3.34	0.798
Migration	Ref: No migration in couple, past 6 mo.	-	-	-	-	-	-
	Male migration, female no migration	9.20	1.85–45.81	**0.007**	0.05	0.004–0.65	**0.022**
	Female migration, male no migration	0.80	0.06–10.89	0.868	1.07	0.16–6.94	0.944
	Both male and female migration	1	EMPTY	-	1	EMPTY	-

* Occupational risk categories were collapsed into two categories (informal/formal low-risk and informal high-risk).

## Data Availability

The datasets generated and analyzed during the current study are available from the corresponding author on reasonable request.
