# Supplementary material for: Mobility is Associated with Higher-risk Sexual Partnerships Among Both Men and Women in Co-resident Couples in Rural Kenya and Uganda: A Longitudinal Cohort Study
Source: AIDS Behav. Author manuscript; Available in PMC 2023 May 18. (PMC10129962; doi:10.1007/s10461-022-03878-0)
Supplement: Suppl Table 1 [file NIHMS1874247-supplement-Suppl_Table_1.docx]

**Suppl. Table I: Sex-pooled** **couple-level mobility effects on** **higher-risk and higher risk and concurrent sexual partnerships, 2016-2020 (n=3501).**

| **Variable** | **Category** | **Sex-pooled higher-risk partnership** | | | **Sex-pooled higher-risk and concurrent partnership** | | |
| --- | --- | --- | --- | --- | --- | --- | --- |
|  |  | **aOR** | **95% CI** | ***p*** | **aOR** | **95% CI** | ***p*** |
| Time | Round | 0.71 | 0.51 - 1.12 | 0.056 | 0.49 | 0.33 – 0.71 | **<0.001** |
| Age | Mean age | 0.98 | 0.94 - 1.02 | 0.260 | 1.03 | 1.00 – 1.07 | 0.070 |
| Education | Ref: No education or some primary | - | - | - | - | - | - |
|  | Completed primary and higher | 0.85 | 0.26 - 2.74 | 0.787 | 4.58 | 1.18 – 17.75 | **0.028** |
| Occupation* | Ref: Formal and informal sector low-risk | - | - | - | - | - | - |
|  | Informal sector high-risk | 6.39 | 2.02 - 20.19 | **0.002** | 5.84 | 1.22 – 27.91 | **0.027** |
| Household wealth | Ref: All other quartiles | - | - | - | - | - | **-** |
|  | Poorest quartile | 3.00 | 0.98 - 9.18 | 0.054 | 11.21 | 2.64 – 47.48 | **0.001** |
| Mobility | Ref: No mobility in couple, past 6 mo. | - | - | - | - | - | - |
|  | Male mobile, female not | 1.08 | 0.38 – 3.05 | 0.885 | 0.81 | 0.38 – 1.74 | 0.591 |
|  | Female mobile, male not | 1.46 | 0.57 – 3.69 | 0.428 | 1.30 | 0.78 – 2.12 | 0.314 |
|  | Both male and female mobile | 2.87 | 1.20 – 6.84 | **0.018** | 2.49 | 1.44 – 4.31 | **0.001** |

In this model, mobility measures any mobility or migration. ***** Occupational risk categories were collapsed into two categories (informal/formal low-risk and informal high-risk).
