# Supplementary material for: Mobility is Associated with Higher-risk Sexual Partnerships Among Both Men and Women in Co-resident Couples in Rural Kenya and Uganda: A Longitudinal Cohort Study
Source: AIDS Behav. Author manuscript; Available in PMC 2023 May 18. (PMC10129962; doi:10.1007/s10461-022-03878-0)
Supplement: Suppl Table 2 [file NIHMS1874247-supplement-Suppl_Table_2.docx]

**Suppl. Table II**: C**ouple-level mobility and work-related mobility effects on** **higher-risk sexual partnerships in men, 2016-2020 (n=1765).**

| **Variable** | **Category** | **Men: ANY mobility** | | | **Men: Work-related mobility** | | |
| --- | --- | --- | --- | --- | --- | --- | --- |
|  |  | **aOR** | **95% CI** | ***p*** | **aOR** | **95% CI** | ***p*** |
| Time | Round | 0.80 | 0.57 - 1.12 | 0.187 | 0.81 | 0.58 - 1.14 | 0.233 |
| Age | Mean age | 0.99 | 0.96 - 1.02 | 0.541 | 0.99 | 0.96 – 1.03 | 0.695 |
| Education | Ref: No education or some primary | - | - | - | - | - | - |
|  | Completed primary and higher | 0.84 | 0.31 - 2.27 | 0.736 | 0.88 | 0.31 – 2.49 | 0.811 |
| Occupation* | Ref: Formal and informal sector low-risk | - | - | - | - | - | - |
|  | Informal sector high-risk | 2.04 | 0.73 – 5.67 | 0.171 | 2.37 | 0.85 – 6.55 | 0.097 |
| Household wealth | Ref: All other quartiles | - | - | - | - | - | - |
|  | Poorest quartile | 2.15 | 0.68 - 6.79 | 0.193 | 1.99 | 0.63 – 6.29 | 0.242 |
| Mobility | Ref: No mobility in couple, past 6 mo. | - | - | - | - | - | - |
|  | Male mobile, female not | 1.80 | 0.71 – 4.53 | 0.212 | 2.41 | 1.13 – 5.15 | **0.024** |
|  | Female mobile, male not | 1.22 | 0.43 – 3.47 | 0.704 | 1.86 | 0.11 – 32.23 | 0.668 |
|  | Both male and female mobile | 2.64 | 1.05 – 6.59 | **0.038** | 1.05 | 0.15 – 7.55 | 0.961 |

In these models, mobility measures any mobility (left) and work-related mobility (right). ***** Occupational risk categories were collapsed into two categories (informal/formal low-risk and informal high-risk).
